# Supplementary material for: A mass spectrum-oriented computational method for ion mobility-resolved untargeted metabolomics
Source: Nat Commun. 2023 Mar 31;14:1813. doi: 10.1038/s41467-023-37539-0 (PMC10066191; doi:10.1038/s41467-023-37539-0)
Supplement: Supplementary file 1 — Supplementary Information [file 41467_2023_37539_MOESM1_ESM.pdf]

A mass spectrum-oriented computational method for ion mobility-resolved untargeted metabolomics

Mingdu Luo[1,2], Yandong Yin[1], Zhiwei Zhou[1], Haosong Zhang[1,2], Xi Chen[1,2], Hongmiao Wang[1,2], and Zheng-Jiang Zhu[1,3,*]

[1] Interdisciplinary Research Center on Biology and Chemistry, Shanghai Institute of Organic Chemistry, Chinese Academy of Sciences, Shanghai, 200032 P. R. China

[2] University of Chinese Academy of Sciences, Beijing, 100049 P. R. China

[3] Shanghai Key Laboratory of Aging Studies, Shanghai, 201210 P. R. China

Corresponding Author

Correspondence should be addressed to Z.J.Z (jiangzhu@sioc.ac.cn)

List for Supplementary Figures

Supplementary Figure 1. The top-down based dimensionality reduction strategy for 4D peak detection in 4D proteomics and metabolomics.

Supplementary Figure 2. The flowchart of Met4DX workflow.

Supplementary Figure 3. The numbers of MS2 spectra in each cluster in human urine sample replicates in positive (a) and negative (b) modes.

Supplementary Figure 4. 4D peak detection in human urine samples in negative mode.

Supplementary Figure 5. Kynurenic acid identification by 4D match and validation by chemical standard.

Supplementary Figure 6. The overlap of 4D features in different biological samples detected by different software tools.

Supplementary Figure 7. Consistency of peak fidelity results obtained from manual check and bioinformatic tool EVA in NIST human urine samples.

Supplementary Figure 8. The peak fidelity rates of MS-DIAL and MetaboScape checked by EVA.

Supplementary Figure 9. The quantification precision of 4D peak detection in negative mode.

Supplementary Figure 10. Feature intensity correlations in MetaboScape.

Supplementary Figure 11. Normalized intensity ratio over the dilution series of 20 natural products in NIST human urine samples.

Supplementary Figure 12. The 4D metabolite library in Met4DX (a) and the confidence levels of metabolite annotations in Met4DX (b).

Supplementary Figure 13. The number of identified features in other biological samples in positive (a) and negative mode (b).

Supplementary Figure 14. Differentiation of isomeric metabolites with the same formula in 4D metabolite library with IM separation, LC separation, and LC×IM dual separations.

Supplementary Figure 15. The number of co-eluting isobaric metabolic feature pairs detected by different software tools.

Supplementary Figure 16. The bottom-up assembly based 4D peak detection in Met4DX enables to detect the isobaric feature pairs of M206T169C147 and M206T166C155 in negative mode of mouse liver samples.

Supplementary Figure 17. The bottom-up assembly based 4D peak detection in Met4DX enables to detect isobaric feature pairs of M692T122C236 and M692T122C242 in negative mode of mouse liver samples.

Supplementary Figure 18. The bottom-up assembly based 4D peak detection in Met4DX enables to detect isobaric feature pairs of M287T291C164 and M287T290C168 in positives mode of mouse liver samples.

Supplementary Figure 19. The bottom-up assembly based 4D peak detection in Met4DX enables to detect isobaric feature pairs of M602T209C235 and M602T208C238 in positives mode of mouse liver samples.

Supplementary Figure 20. LC×IM dual separation in LC–IM–MS-based metabolomics provided better resolving power for co-eluting isobaric feature pairs.

Supplementary Figure 21. Detection and discrimination of co-eluting isobaric feature pairs shown in Figure 5f-i using MS-DIAL (a-d) and MetaboScape (e-h).

Supplementary Figure 22. The example of co-eluting isobaric features detected by Met4DX with small CCS difference but separated with LC×IM dual separations.

Supplementary Figure 23. LC separation in multidimensional separation in LC–IM–MS.

Supplementary Figure 24. Metabolite annotations of co-eluted isobaric features M206T168C147 and M206T166C155.

Supplementary Figure 25. A chimeric MS2 spectrum acquired for the isomeric metabolites of N-acetyl-L-phenylalanine and 3-phenylpropionylglycine in mouse liver samples acquired on LC-MS (negative mode) without IM separation.

Supplementary Figure 26. Met4DX supports peak detection using precursor ions retrieved from MS2 spectra and a user inputted ions of interest list.

Supplementary Figure 27. The Met4DX workflow using a user-inputted list of precursor ions.

Supplementary Figure 28. High-coverage and high quantification precision of 4D peak detection in Met4DX using the inputted precursor ion list.

Supplementary Figure 29. 4D peaks obtained from Met4DX showed higher quantification quality and MS2 spectral coverage than those from MS-DIAL.

Supplementary Figure 30. Met4DX enabled high-coverage 4D peak detection and MS2 spectral extraction in PASEF-DIA data compared with other software tools.

Supplementary Figure 31. The processing of IM-AIF metabolomics data acquired using Agilent DTIM-MS instrument using Met4DX and MS-DIAL.

Supplementary Figure 32. Validations of RT prediction errors in the transfer learning models.

Supplementary Figure 33. Cumulative percentages of predicted CCS values of external validation sets in AllCCS.

Supplementary Figure 34. Met4DX showed high consistency results in peak detection using MS2 spectra converted from Bruker DataAnalysis software and generated by the “GenerateMS2” function in Met4DX.

List for Supplementary Table

Supplementary Table 1: Parameter set to run Met4DX (PASEF-DDA, MS2 spectra oriented).

Supplementary Table 2: Parameter set to run Met4DX (PASEF-DDA, precursor ion list).

Supplementary Table 3: Parameter set to run Met4DX (PASEF-DIA, precursor ion list).

Supplementary Table 4: Parameter set to run Met4DX (IM-AIF, precursor ion list).

Supplementary Table 5: Parameter set to run MS-DIAL.

Supplementary Table 6: Parameter set to run MetaboScape.

List for Supplementary Note

Supplementary Note 1: Instruction to perform metabolite annotation with user-inputted library in Met4DX.

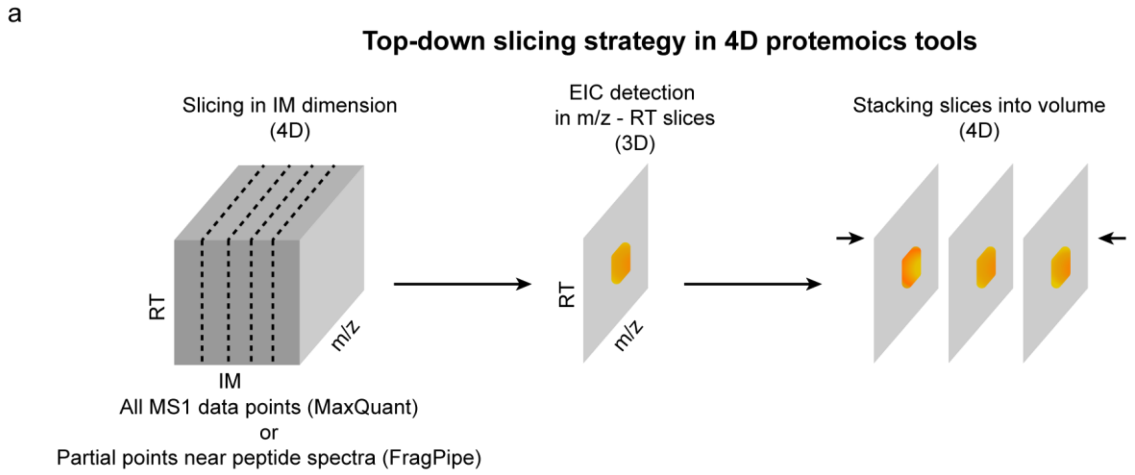

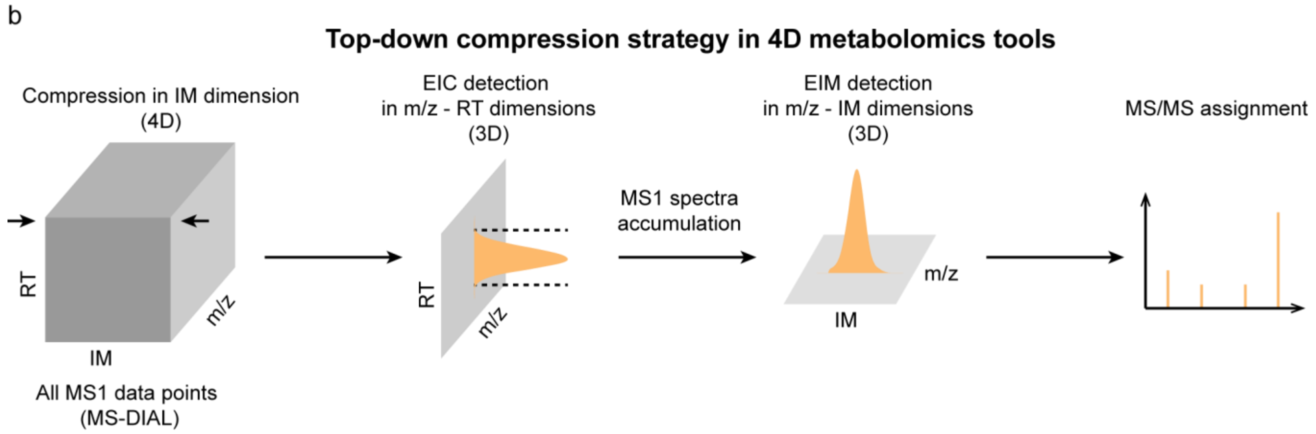

Supplementary Figure 1. The top-down based dimensionality reduction strategy for 4D peak detection in 4D proteomics and metabolomics. (**a**) Top-down slicing strategy in 4D proteomics software tools, such as MaxQuant and FragPipe; (**b**) Top-down compression strategy in 4D metabolomics software tools, such as MS-DIAL.

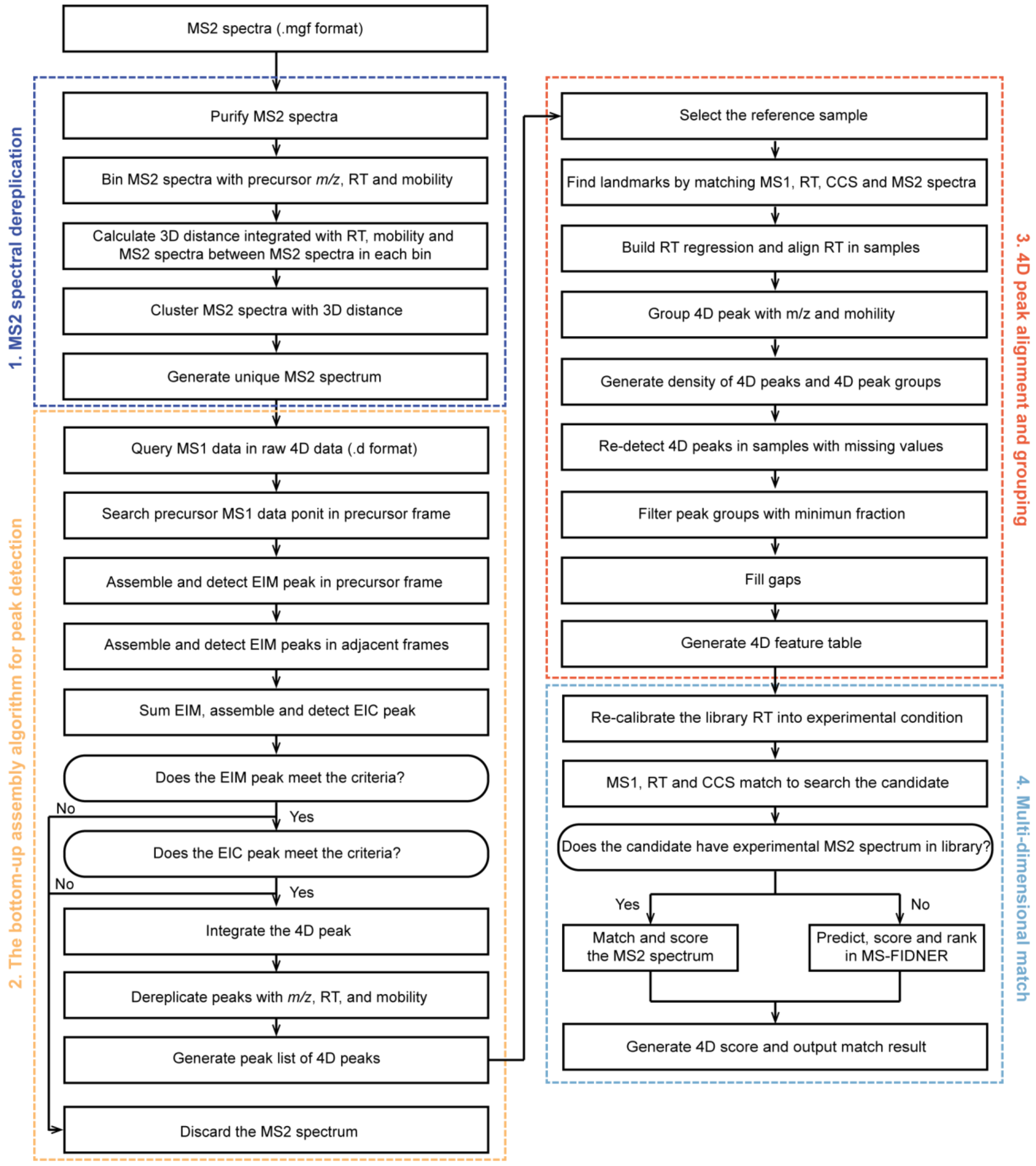

Supplementary Figure 2. The flowchart of Met4DX workflow.

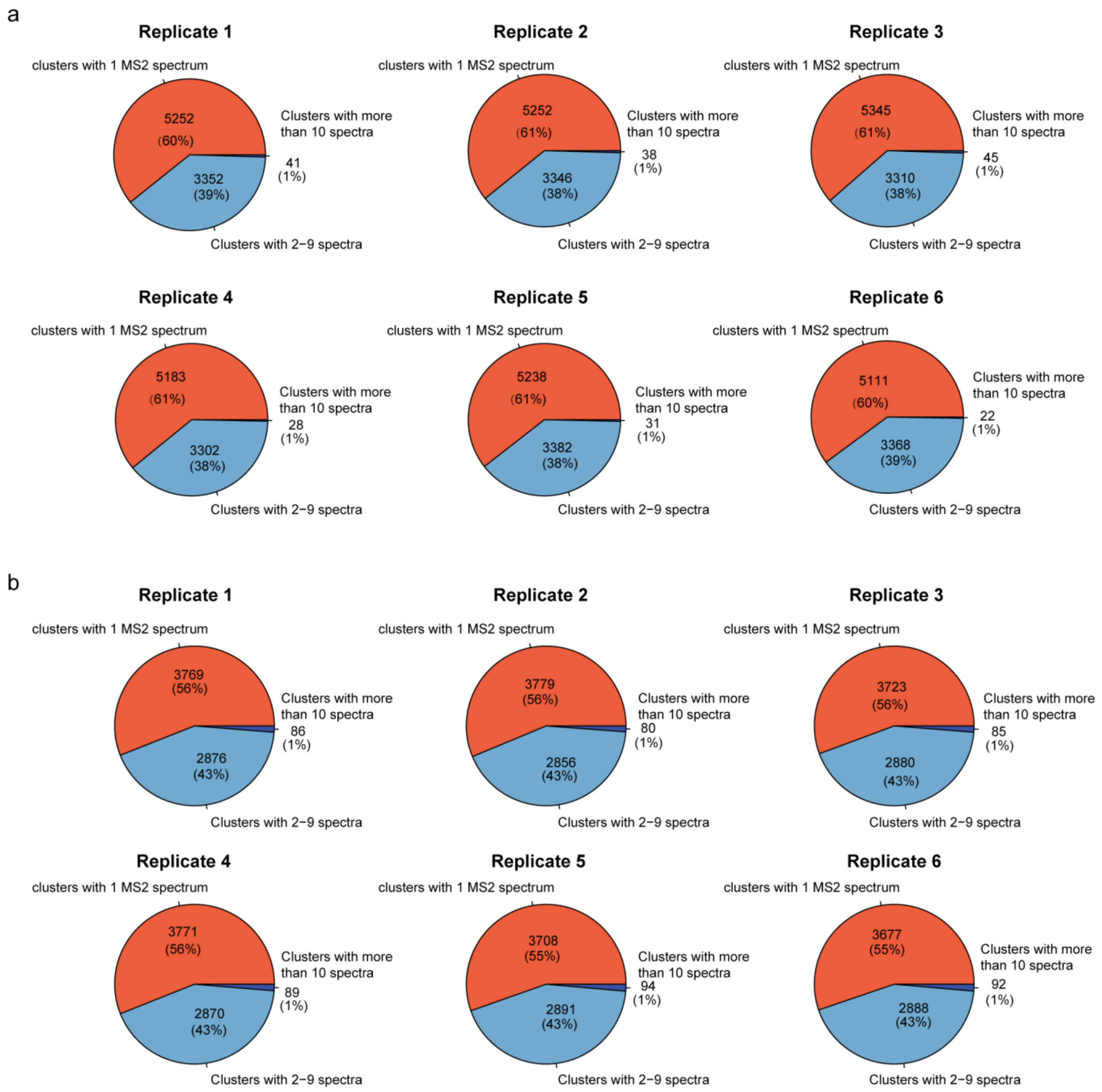

Supplementary Figure 3. The numbers of MS2 spectra in each cluster in human urine sample replicates in positive (**a**) and negative (**b**) modes.

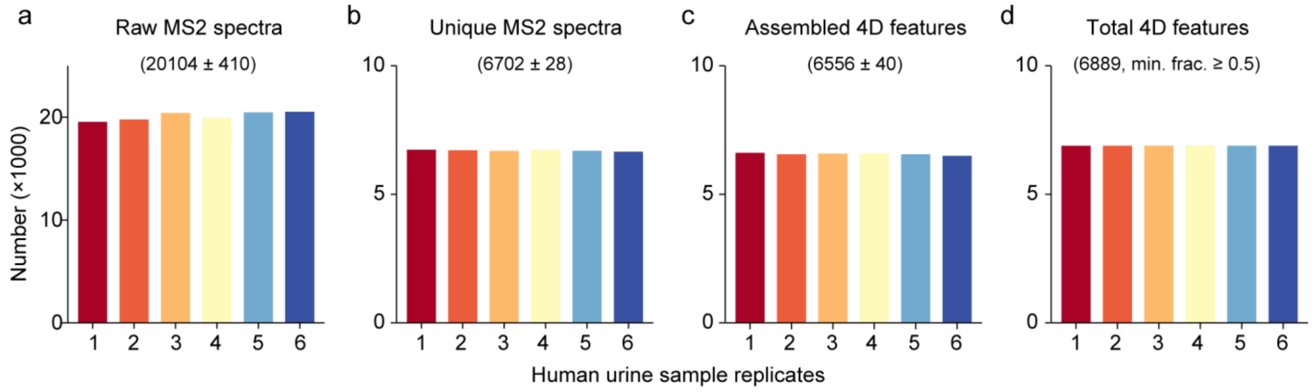

Supplementary Figure 4. 4D peak detection in human urine samples in negative mode. (**a-c**) The numbers of raw MS2 spectra (**a**), unique MS2 spectra (**b**), and assembled 4D features (**c**) in each sample replicate. Numbers represent mean±SD (n= 6 technical replicates). (**d**) The total number of 4D features with a minimal fraction of 0.5 applied (minimal fraction ≥ 0.5).

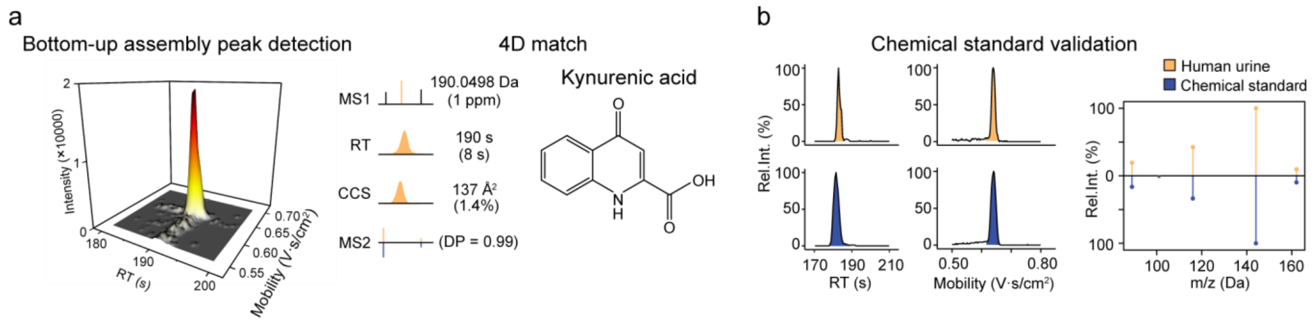

Supplementary Figure 5. Kynurenic acid identification by 4D match and validation by chemical standard. (**a**) The 4D match result of kynurenic acid by Met4DX. (**b**) The chemical standard validation of kynurenic acid.

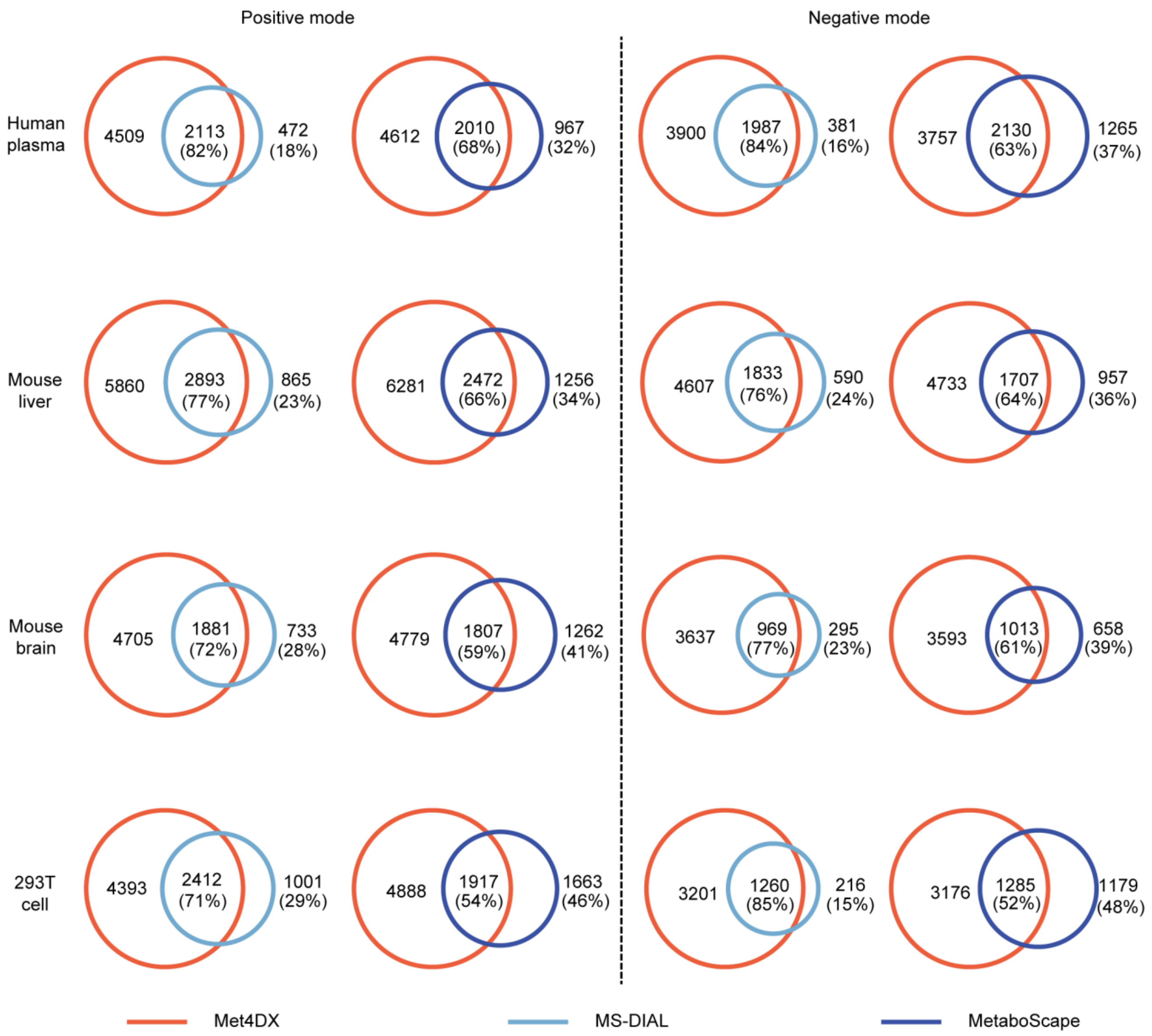

Supplementary Figure 6. The overlap of 4D features in different biological samples detected by different software tools.

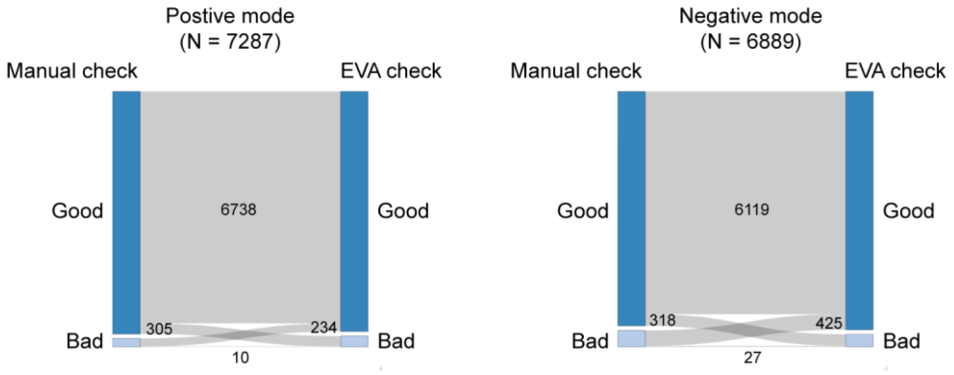

Supplementary Figure 7. Consistency of peak fidelity results obtained from manual check and bioinformatic tool EVA in NIST human urine samples (PASEF-DDA; positive and negative modes).

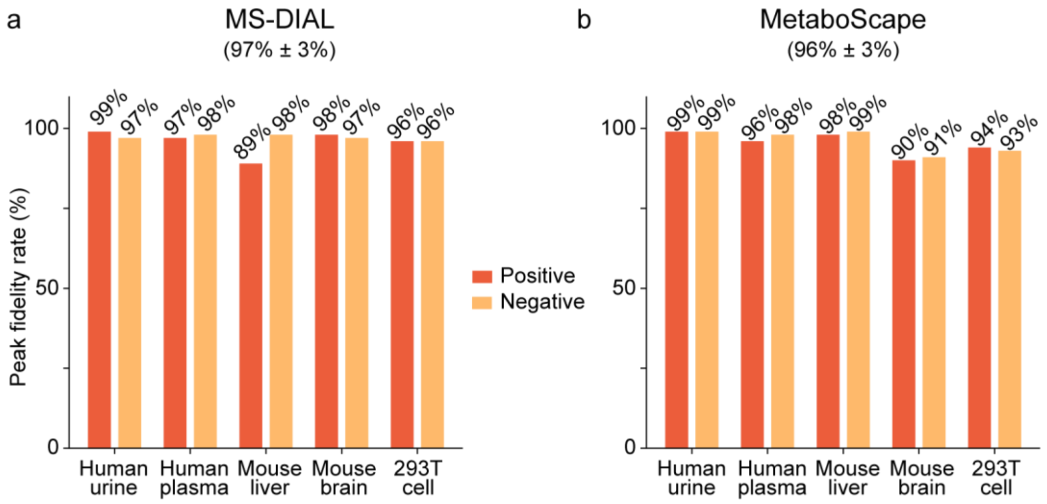

Supplementary Figure 8. The peak fidelity rates of MS-DIAL and MetaboScape checked by EVA. The EIC and EIM peaks obtained from MS-DIAL (**a**) and MetaboScape (**b**) were plotted and the peak fidelity were evaluated by EVA.

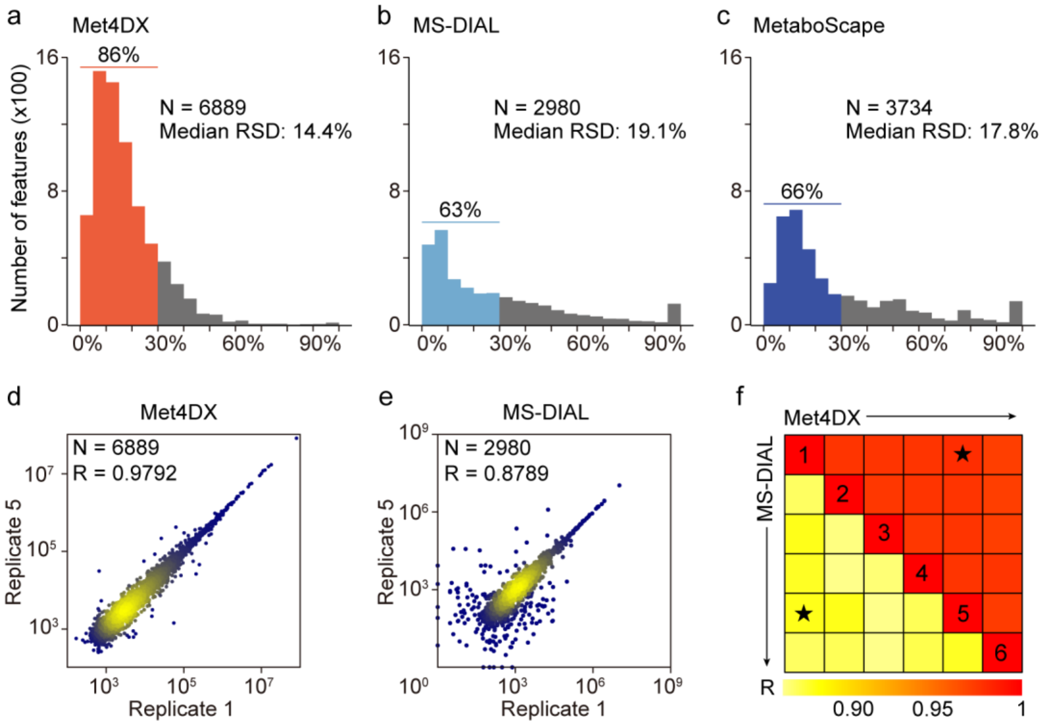

Supplementary Figure 9. The quantification precision of 4D peak detection in negative mode. (**a-c**) The distributions of RSDs of features from human urine samples in negative mode detected by Met4DX (**a**), MS-DIAL (**b**), and MetaboScape (**c**). (**d-e**) Log-log feature intensity correlations in Met4DX (**d**) and MS-DIAL (**e**) of the same pair of human urine replicates in negative mode. The color represents the feature density, in which yellow means dense and blue means sparse. (**f**) All pairwise Pearson correlations of feature intensities in 6 replicates of human urine samples in negative mode using Met4DX (upper triangle) and MS-DAL (lower triangle).

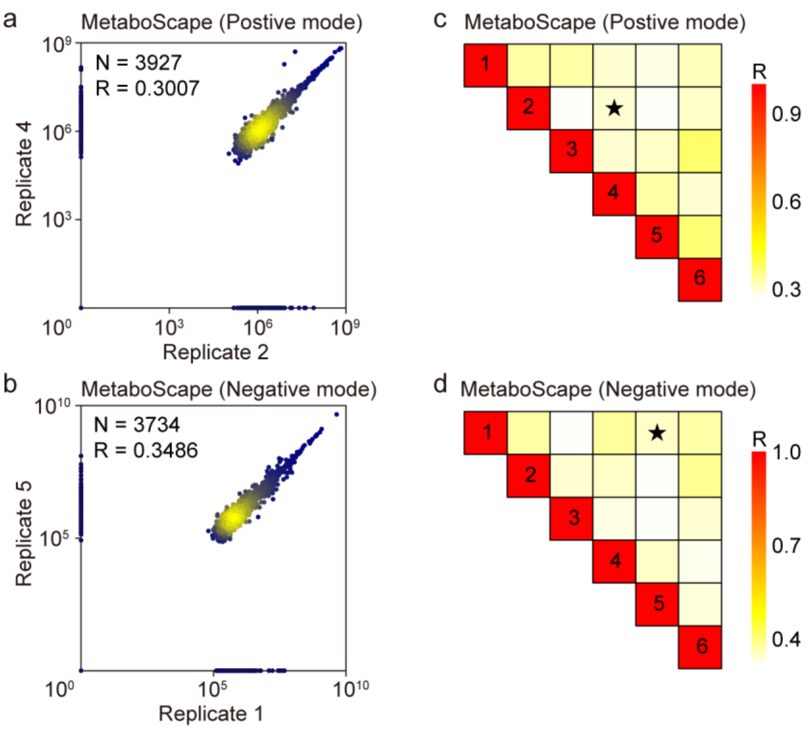

Supplementary Figure 10. Feature intensity correlations in MetaboScape. (**a-b**) Log-log feature intensity correlations a pair of human urine replicates in positive mode (**a**) and negative mode (**b**). The color represents the feature density, in which yellow means dense and blue means sparse. (**c-d**) All pairwise Pearson correlations of feature intensities in 6 replicates of human urine samples using MetaboScape in positive mode (**c**) and negative mode (**d**).

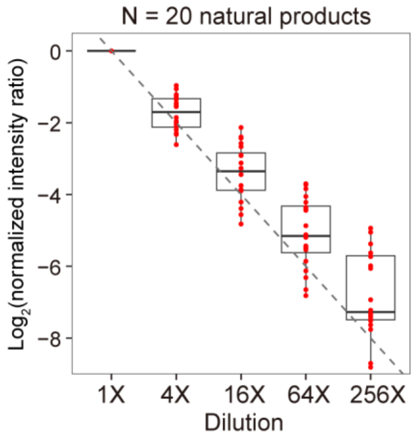

Supplementary Figure 11. Normalized intensity ratio over the dilution series of 20 natural products in NIST human urine samples. Each sample was acquired with 6 technical replicates and the median peak areas of each natural product under one dilution condition were normalized by the median peak area of its highest concentration. The dashed line indicates the expected four-fold difference along the dilution. The box plots indicate median, 25th and 75th percentiles (middle line, Q1 and Q3 within the box, respectively), including 1.5x interquartile range whiskers and all data points.

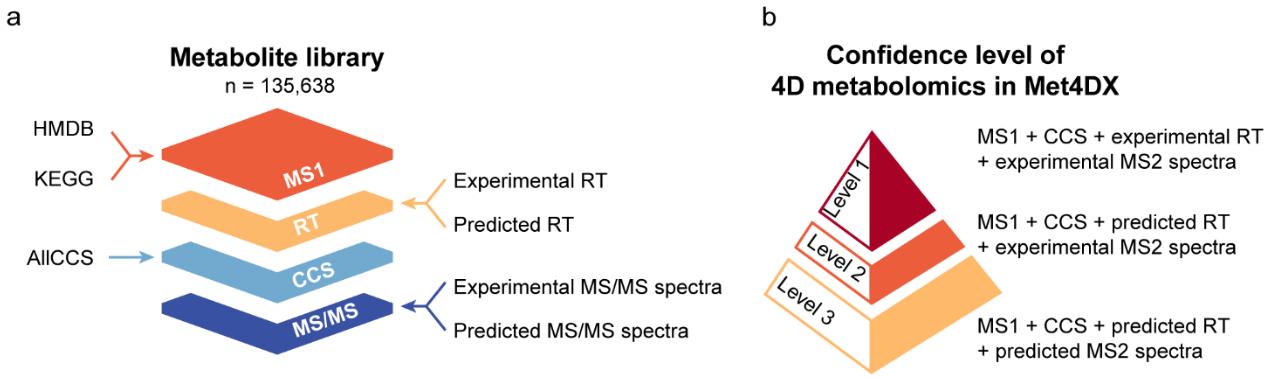

Supplementary Figure 12. The 4D metabolite library in Met4DX (**a**) and the confidence levels of metabolite annotations in Met4DX (**b**).

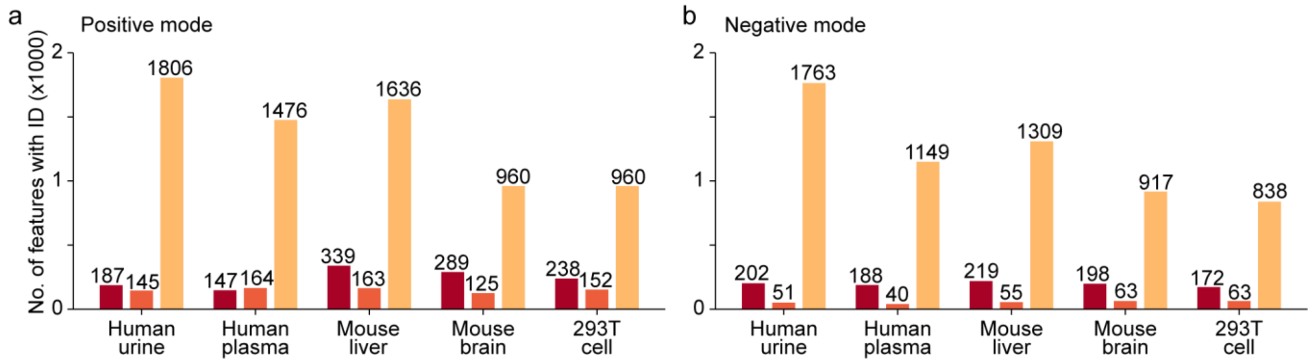

Supplementary Figure 13. The number of identified features in other biological samples in positive (**a**) and negative mode (**b**).

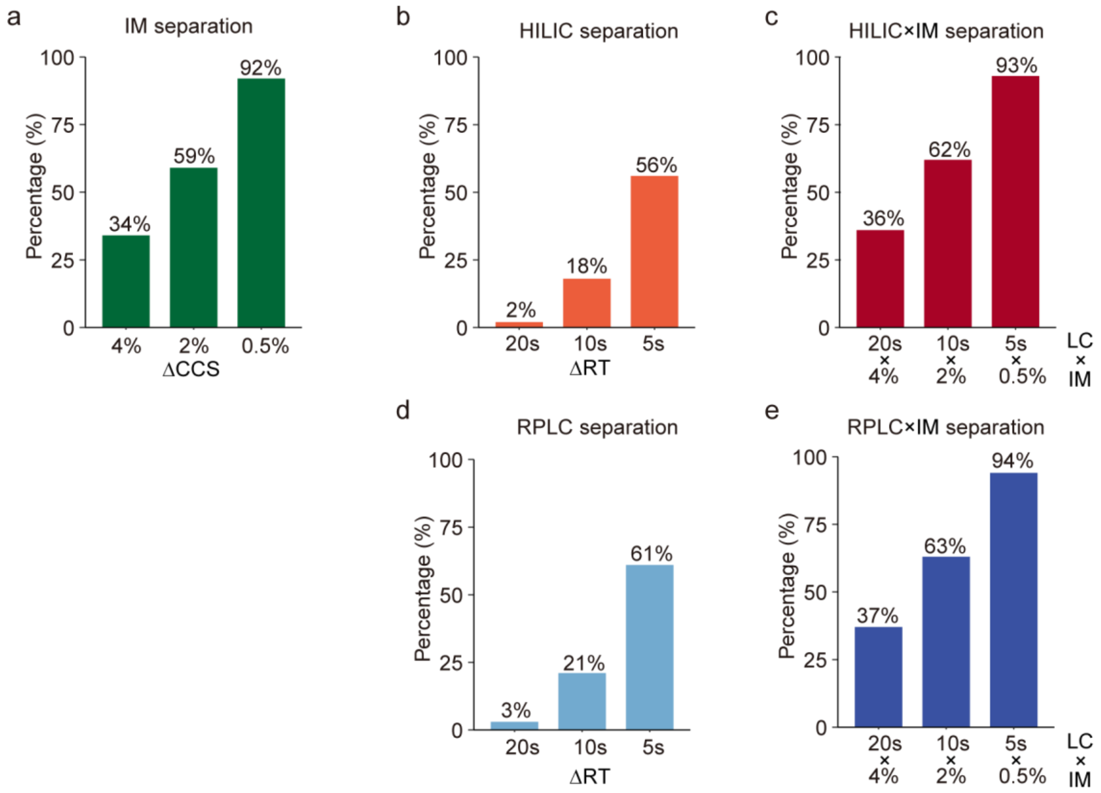

Supplementary Figure 14. Differentiation of isomeric metabolites with the same formula in 4D metabolite library (N=16,484,071 pairs) with IM separation (**a**), LC separation (**b** for HILIC and **d** for RPLC) and LC×IM dual separations (**c** for HILIC×IM and **e** for RPLC×IM).

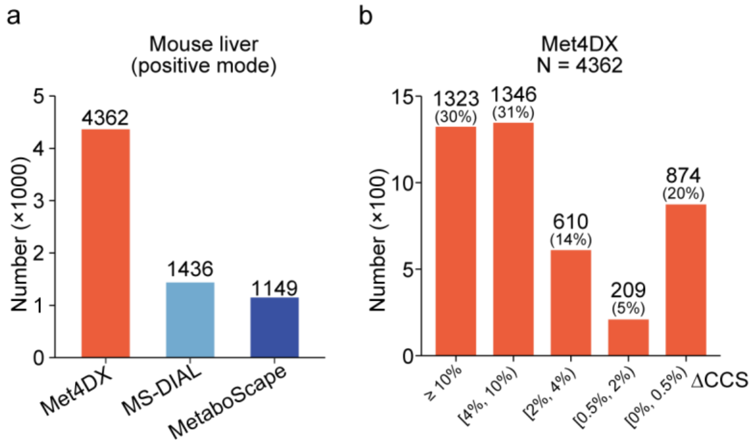

Supplementary Figure 15. The number of co-eluting isobaric metabolic feature pairs detected by different software tools. (**a**) The number of co-eluting isobaric metabolic feature pairs detected in different software tools in positive mode of mouse liver samples (n = 6 technical replicates). (**b**) The CCS difference of co-eluting isobaric features discriminated by Met4DX in positive mode of mouse liver samples.

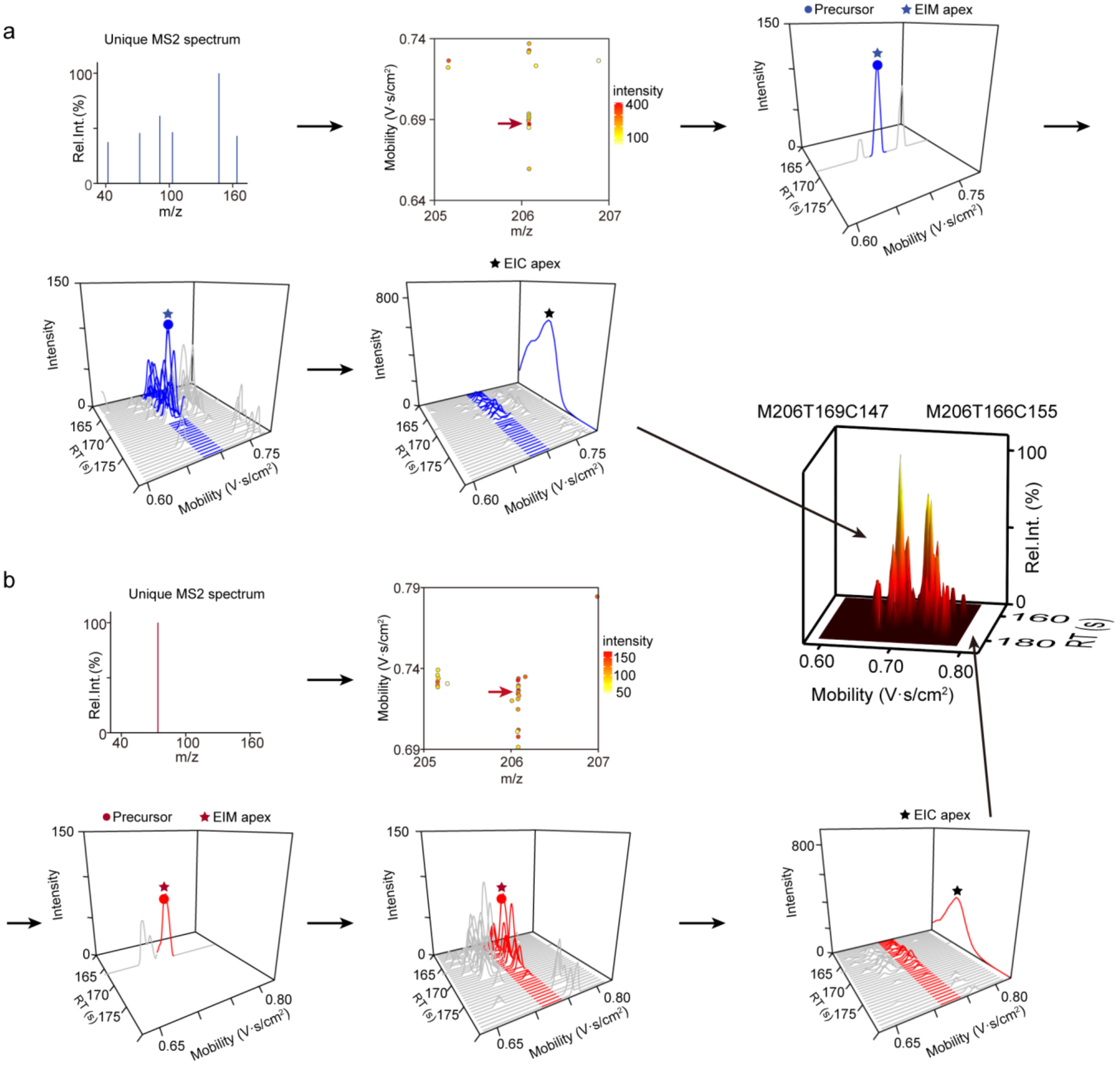

Supplementary Figure 16. The bottom-up assembly based 4D peak detection in Met4DX enables to detect the isobaric feature pairs of M206T169C147 and M206T166C155 in negative mode of mouse liver samples. (**a**) Peak detection of M206T166C155. (**b**) Peak detection of M206T166C155.

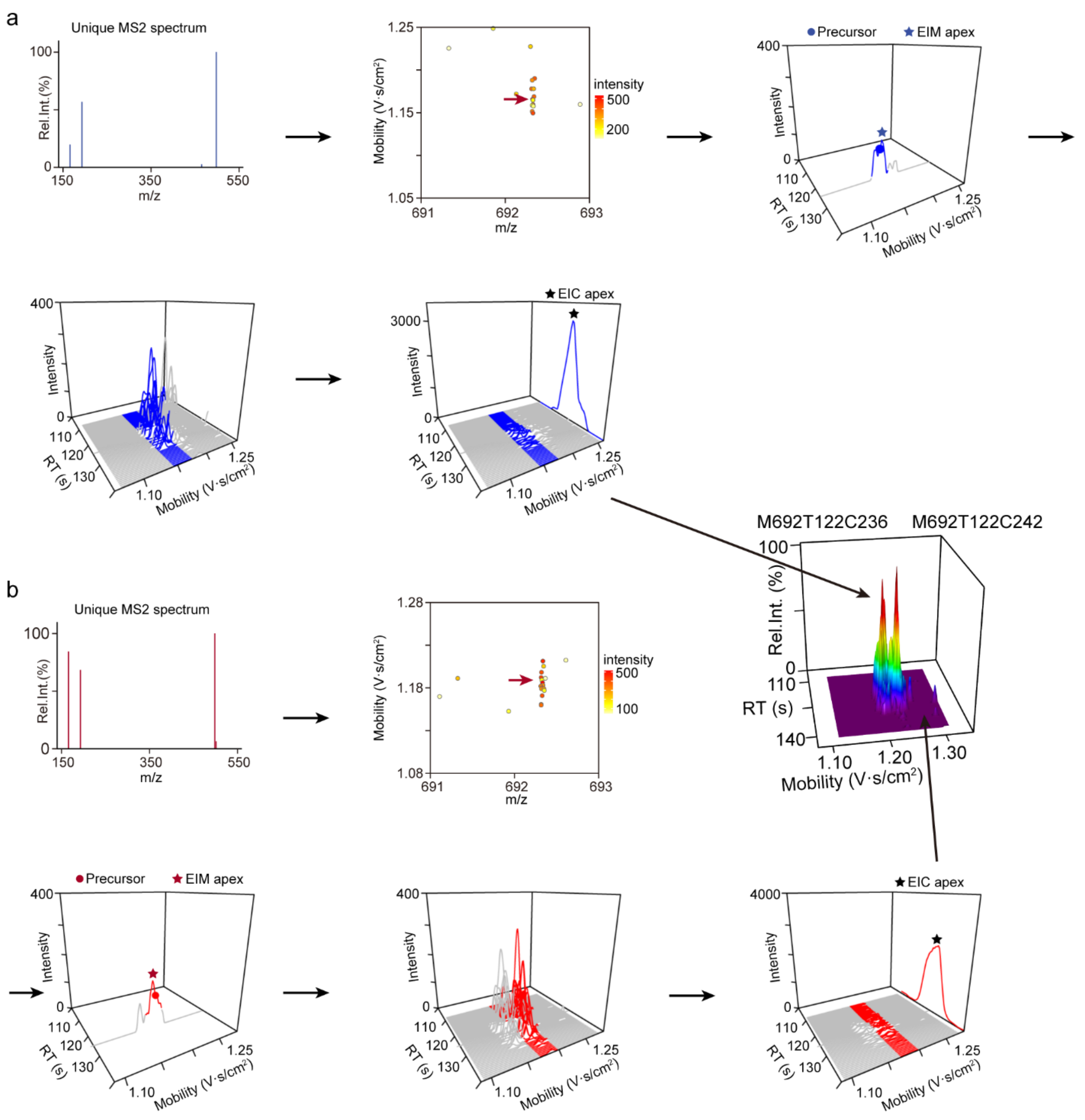

Supplementary Figure 17. The bottom-up assembly based 4D peak detection in Met4DX enables to detect isobaric feature pairs of M692T122C236 and M692T122C242 in negative mode of mouse liver samples. (**a**) Peak detection of M692T122C236. (**b**) Peak detection of M692T122C242.

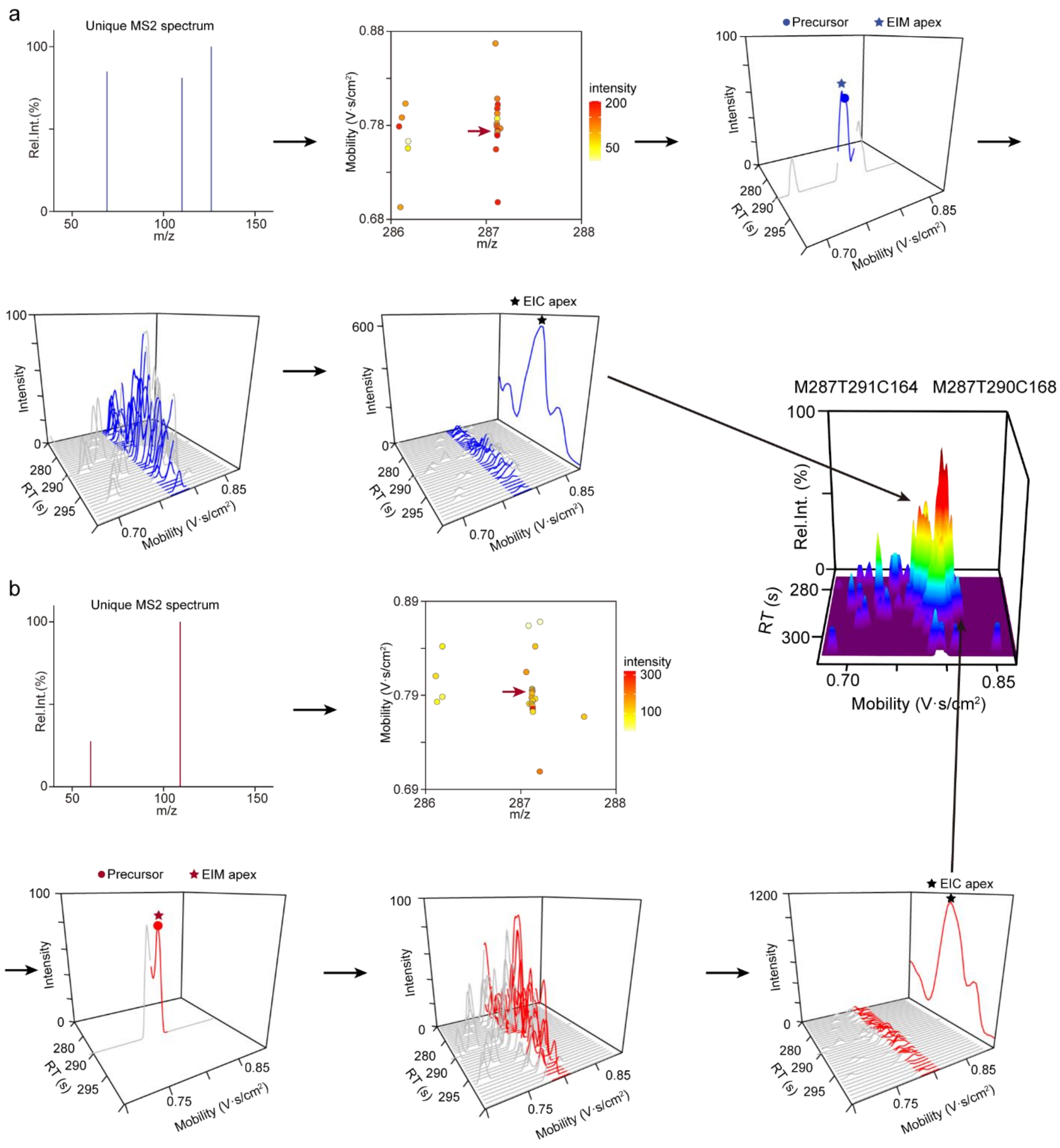

Supplementary Figure 18. The bottom-up assembly based 4D peak detection in Met4DX enables to detect isobaric feature pairs of M287T291C164 and M287T290C168 in positive mode of mouse liver samples. (**a**) Peak detection of M287T291C164. (**b**) Peak detection of M287T290C168.

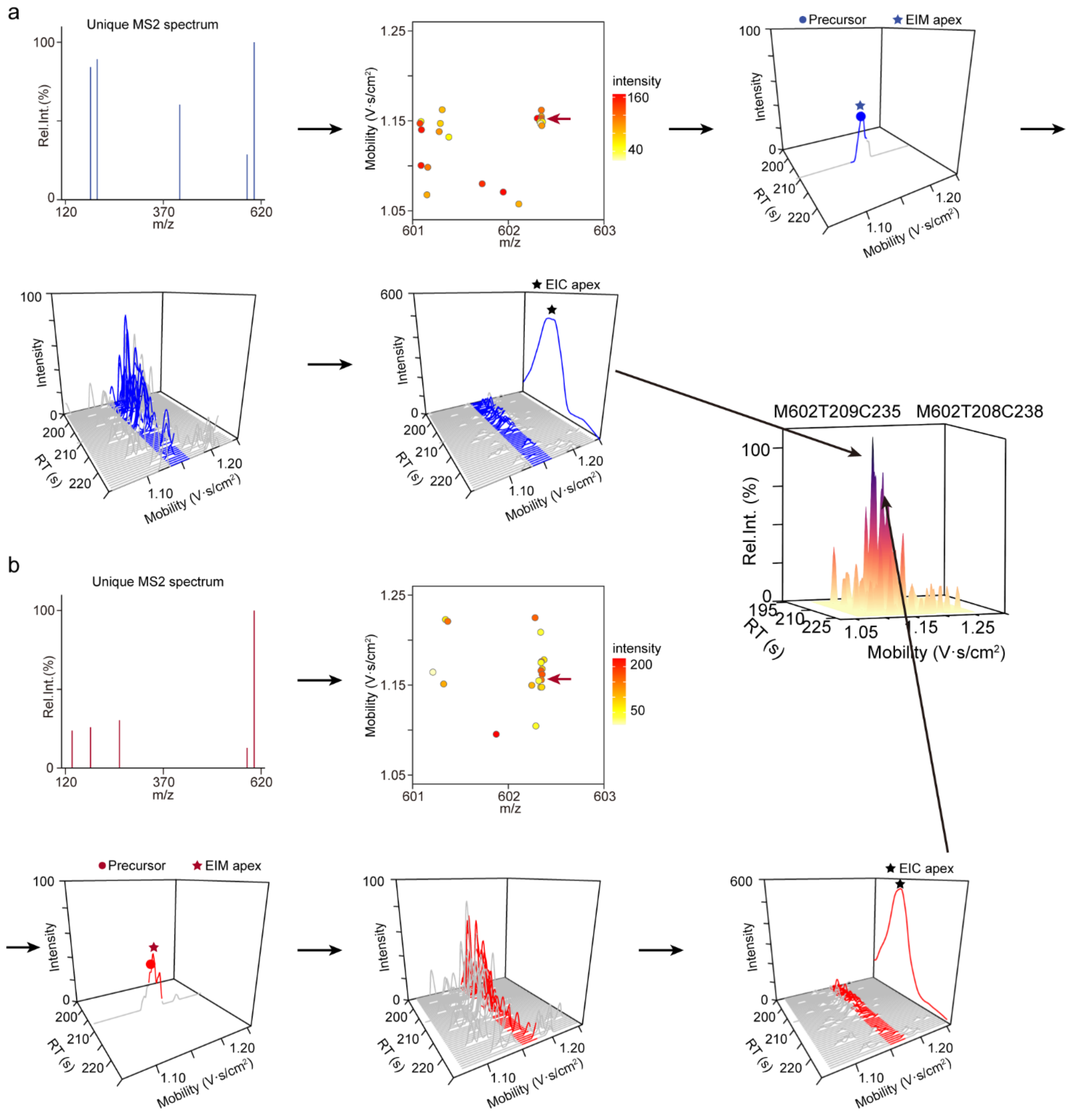

Supplementary Figure 19. The bottom-up assembly based 4D peak detection in Met4DX enables to detect isobaric feature pairs of M602T209C235 and M602T208C238 in positive mode of mouse liver samples. (**a**) Peak detection of M602T209C235. (**b**) Peak detection of M602T209C235.

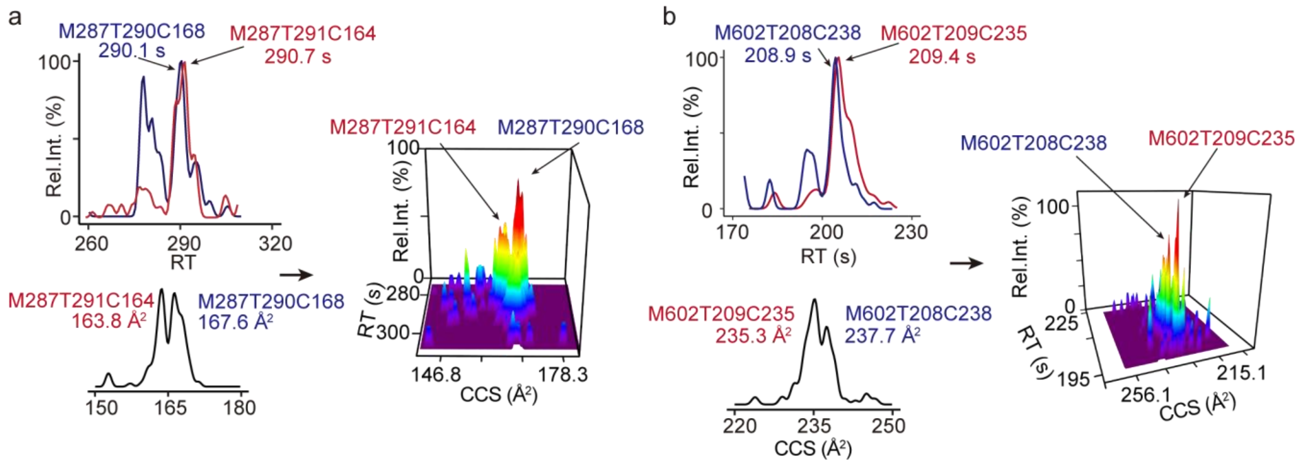

Supplementary Figure 20. LC×IM dual separation in LC–IM–MS-based metabolomics provided better resolving power for co-eluting isobaric feature pairs.

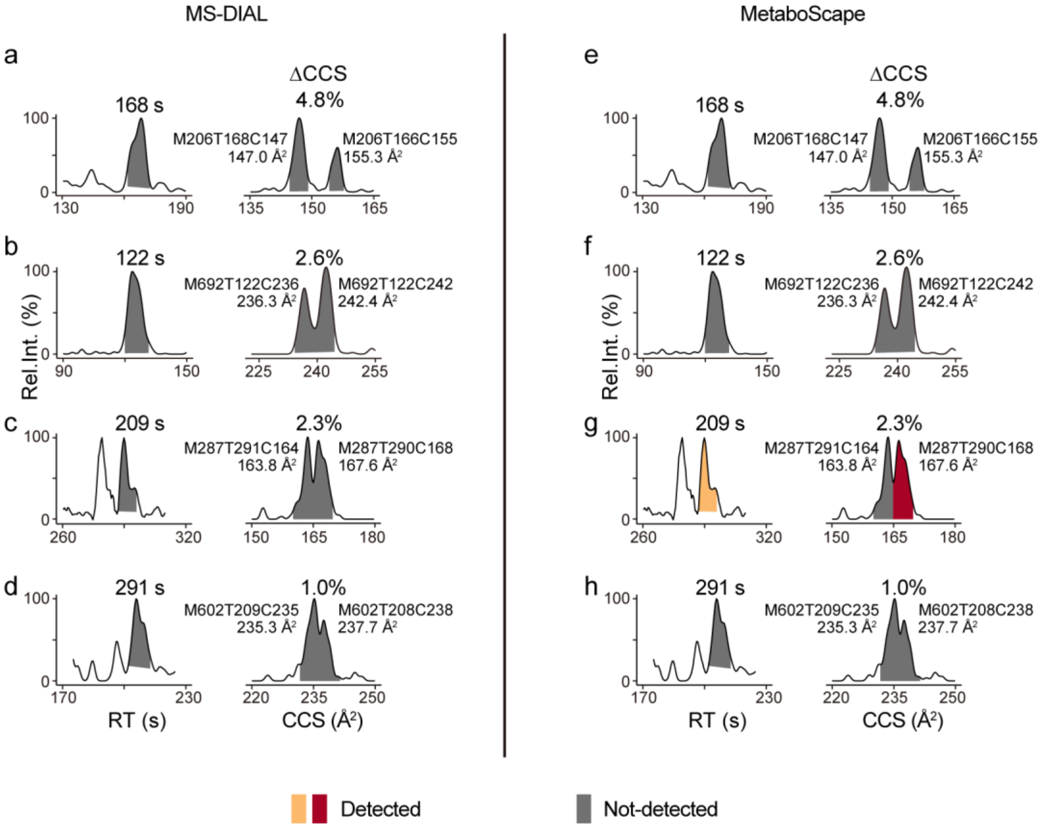

Supplementary Figure 21. Detection and discrimination of co-eluting isobaric feature pairs shown in Figure 5f-i using MS-DIAL (**a-d**) and MetaboScape (**e-h**).

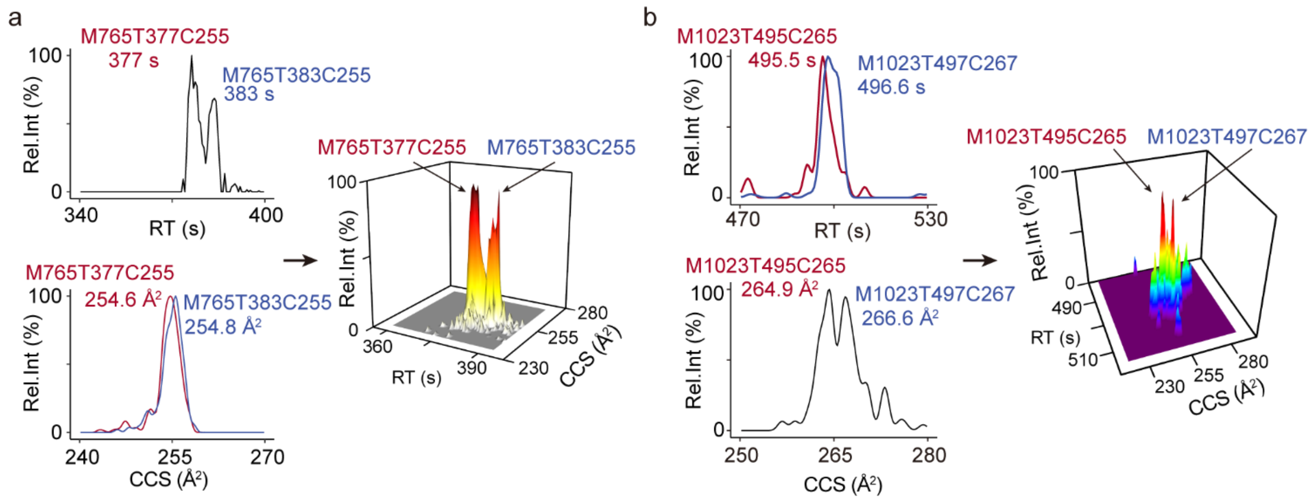

Supplementary Figure 22. The example of co-eluting isobaric features detected by Met4DX with small CCS difference but separated with LC×IM dual separations.

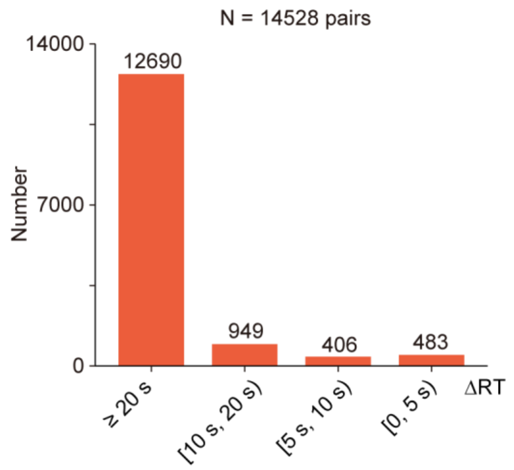

Supplementary Figure 23. The distributions of ΔRT for isobaric feature pairs in multidimensional metabolomic analysis using LC–IM–MS. Isobaric feature pairs with Δm/z ≤ 10 ppm and ΔCCS ≤ 2% were used for statistics here. The data was from mouse liver tissue samples.

	N-acetyl-L-phenylalanine_[M-H]-<br>m/z = 206.0822 Da; RT = 171 s; CCS = 146.7 Å²			3-phenylpropionylglycine_[M-H]-<br>m/z = 206.0822 Da; RT = 170 s; CCS = 155.0 Å²		
**M206T168C147**<br>m/z = 206.0828 Da<br>RT = 168 s<br>CCS = 147.0 Å²	RT error<br>3s<br>RT score<br>1	CCS error<br>0.2%<br>CCS score<br>1	MS2 score<br>0.83<br>**Final score**<br>**0.93**	RT error<br>2s<br>RT score<br>1	CCS error<br>5.1%<br>CCS score<br>0.28	MS2 score<br>0.05<br>**Final score**<br>**0.33**
**M206T166C155**<br>m/z = 206.0826 Da<br>RT = 166 s<br>CCS = 155.3 Å²	RT error<br>5s<br>RT score<br>1	CCS error<br>5.8%<br>CCS score<br>0.05	MS2 score<br>0<br>**Final score**<br>**0.22**	RT error<br>4s<br>RT score<br>1	CCS error<br>0.2%<br>CCS score<br>1	MS2 score<br>0.99<br>**Final score**<br>**0.99**

Supplementary Figure 24. Metabolite annotations of co-eluted isobaric features M206T168C147 and M206T166C155.

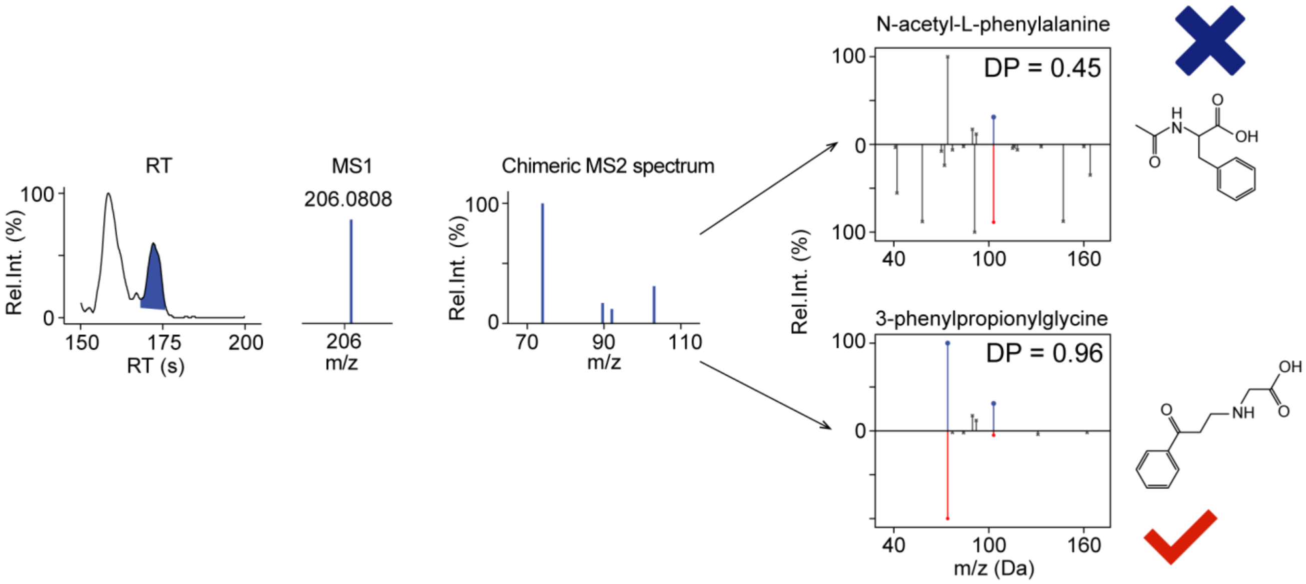

Supplementary Figure 25. A chimeric MS2 spectrum acquired for the isomeric metabolites of N-acetyl-L-phenylalanine and 3-phenylpropionylglycine in mouse liver samples acquired on LC-MS (negative mode) without IM separation.

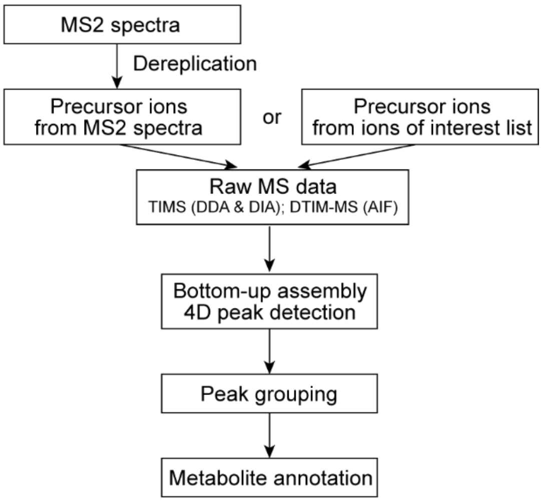

Supplementary Figure 26. Met4DX supports peak detection using precursor ions retrieved from MS2 spectra and a user inputted ions of interest list.

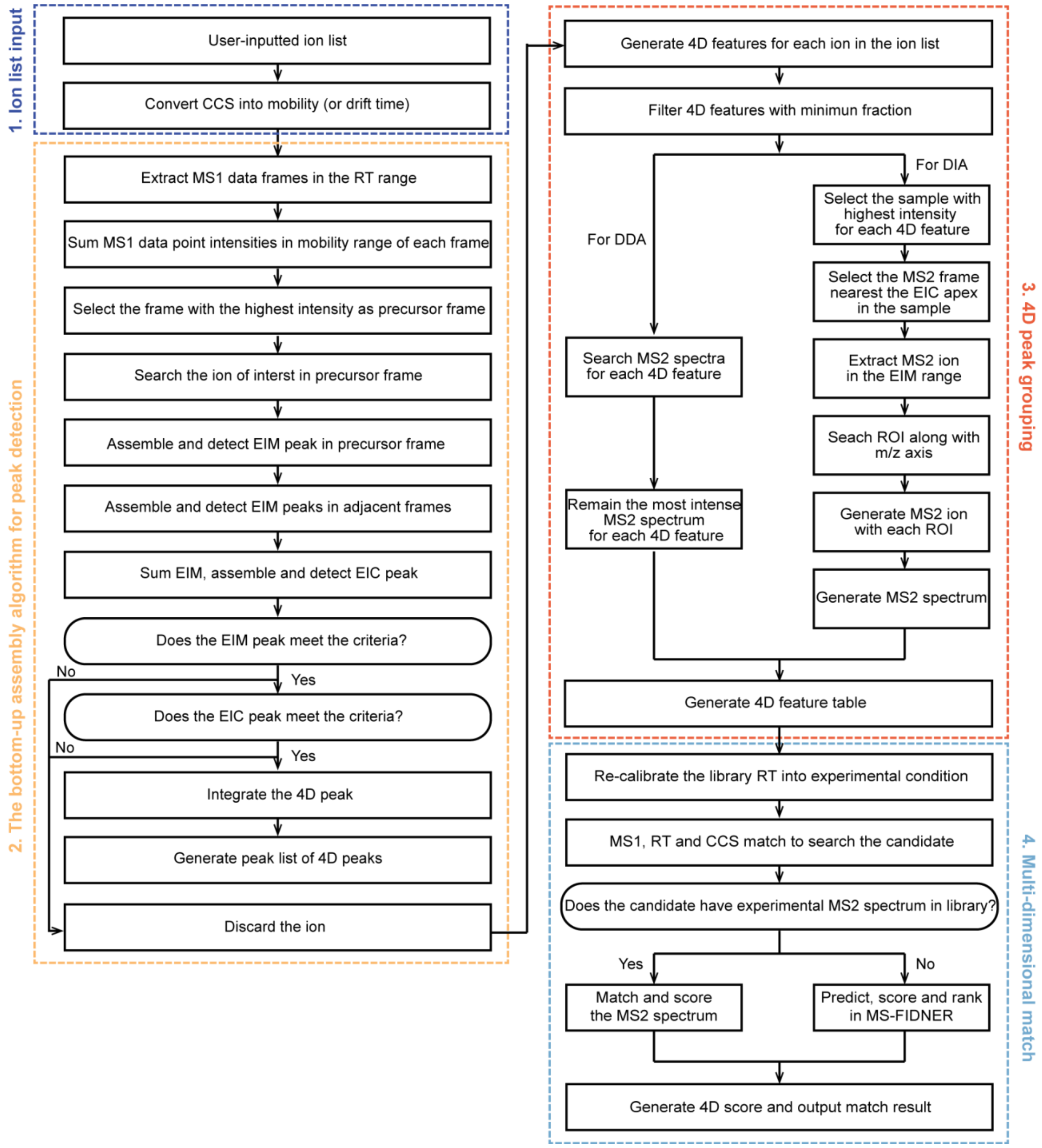

Supplementary Figure 27. The Met4DX workflow using a user-inputted list of precursor ions.

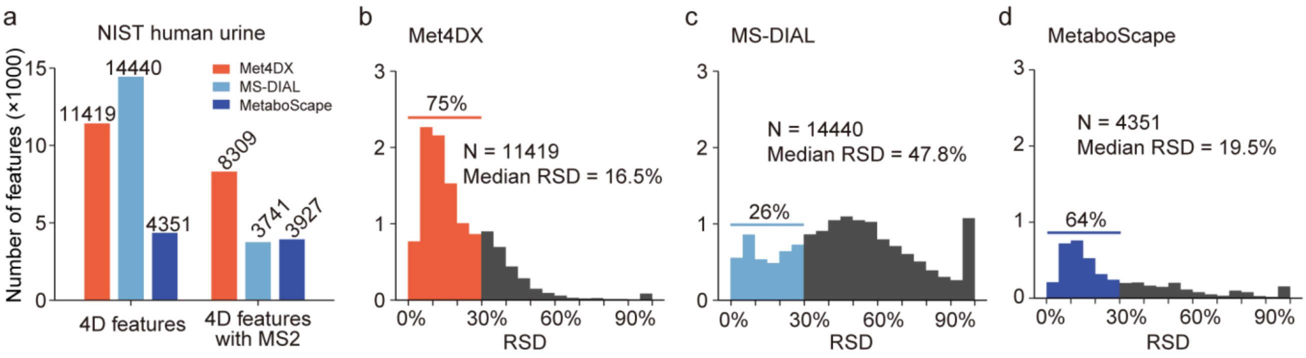

Supplementary Figure 28. High-coverage and high quantification precision of 4D peak detection in Met4DX using the inputted precursor ion list. The data was acquired from NIST human urine samples with PASEF-DDA in positive mode (n=6 technical replicates).

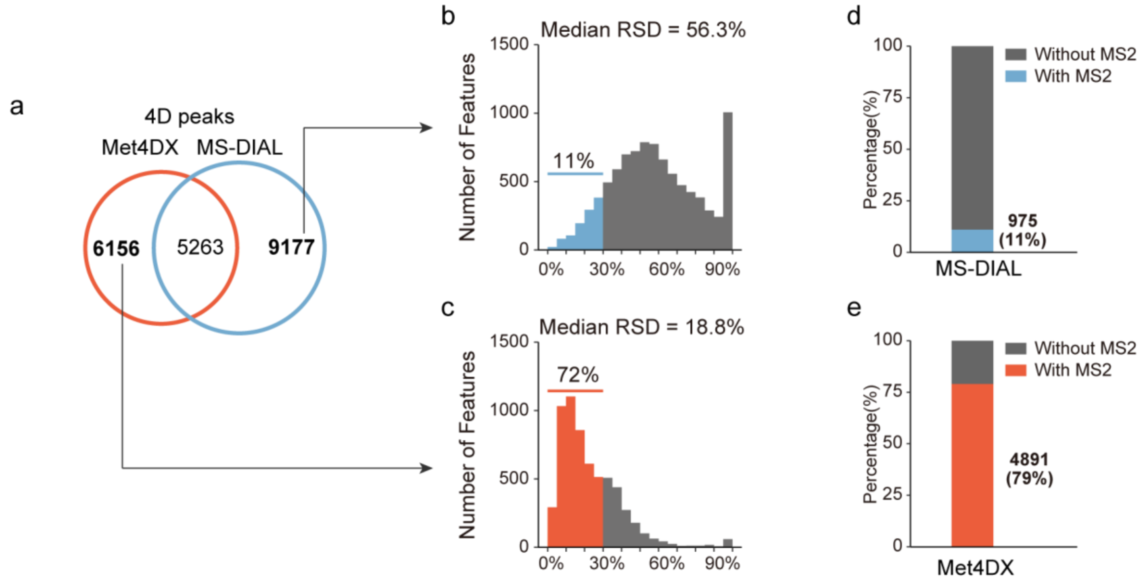

Supplementary Figure 29. 4D peaks obtained from Met4DX showed higher quantification quality and MS2 spectral coverage than those from MS-DIAL. (**a**) Overlaps of 4D peaks obtained from Met4DX and MS-DIAL. (**b-c**) The distributions of relative standard deviation (RSD) of 4D peaks from MS-DIAL (**b**) and Met4DX (**c**). (**d-e**) The MS2 spectral coverage of 4D peaks obtained from MS-DIAL (**d**) and Met4DX (**e**). NIST human urine data (PASEF-DDA; positive mode; n=6 technical replicates) was used here.

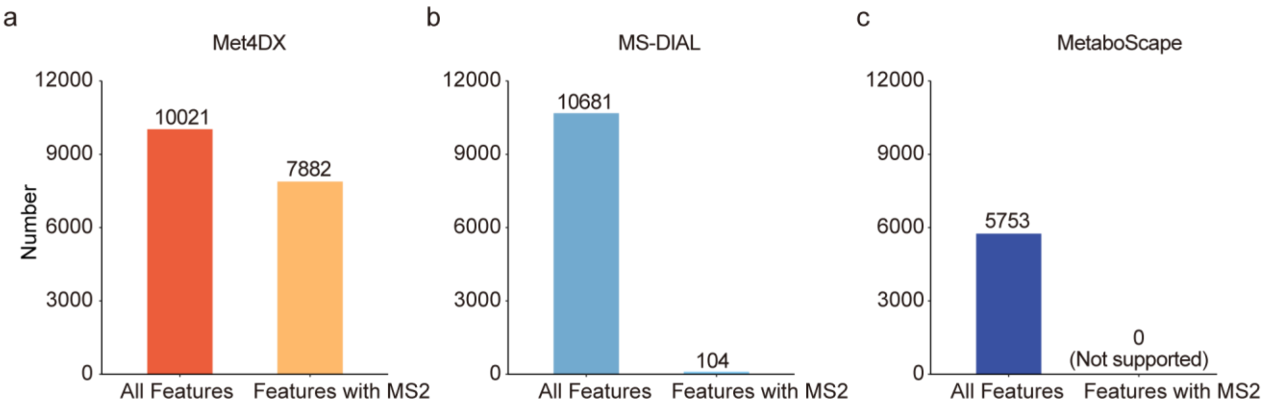

Supplementary Figure 30. Met4DX enabled high-coverage 4D peak detection and MS2 spectral extraction in PASEF-DIA data compared with other software tools. The data was acquired from NIST human urine samples with PASEF-DIA in positive mode (n=6 technical replicates).

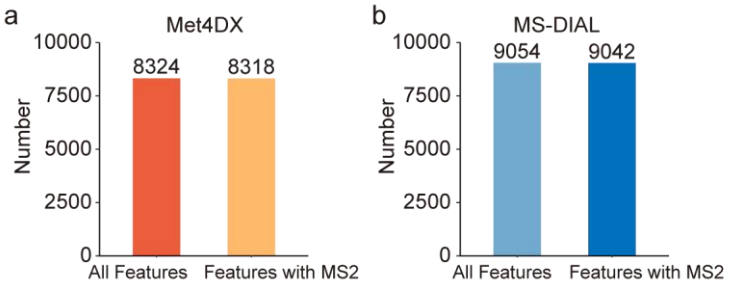

Supplementary Figure 31. The processing of IM-AIF metabolomics data acquired using Agilent DTIM-MS instrument using Met4DX (a) and MS-DIAL (b). The data was acquired from NIST human urine samples in positive mode (n=6 technical replicates).

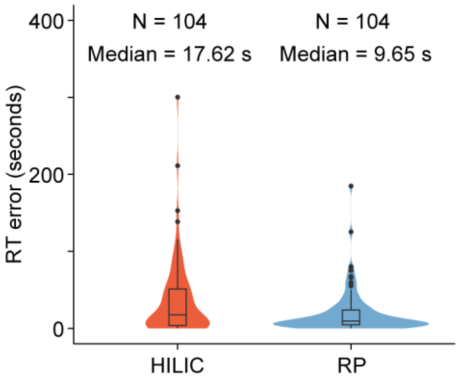

Supplementary Figure 32. Validations of RT prediction errors in the transfer learning models. The box plots indicate median, 25th and 75th percentiles (middle line, Q1 and Q3 within the box, respectively), including 1.5x interquartile range whiskers and outliers (single points outside this range).

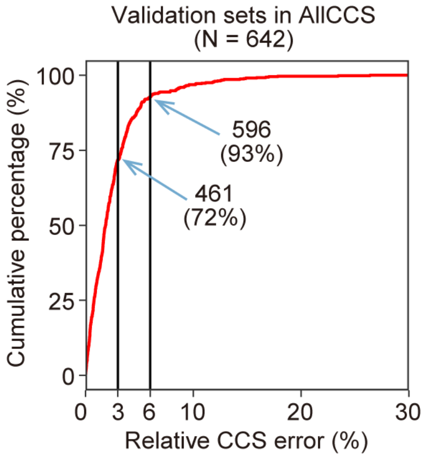

Supplementary Figure 33. Cumulative percentages of predicted CCS values of external validation sets in AllCCS. There were 72% of CCS values with relative errors less than 3% and 93% of CCS values with relative errors less than 6%.

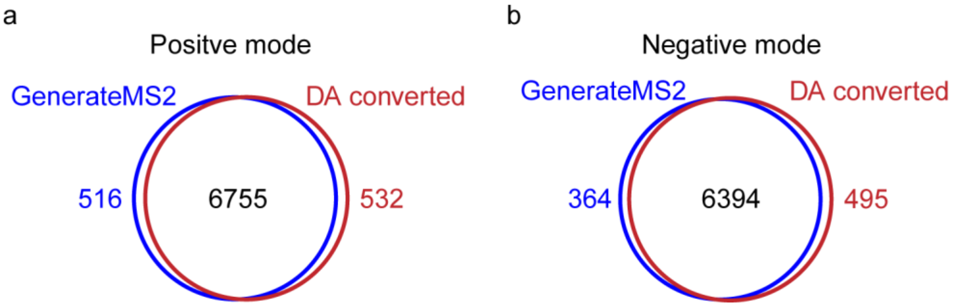

Supplementary Figure 34. Met4DX showed high consistency in peak detection using MS2 spectra converted from Bruker DataAnalysis software (DA converted) and generated by the “GenerateMS2” function in Met4DX. The data was acquired from NIST human urine samples with PASEF-DDA in positive mode (n=6 technical replicates).

Supplementary Table 1. Parameter set to run Me4DX (PASEF-DDA, MS2 spectra oriented).

Parameter	Value
**# MS2 spectral dereplication**	
Absolute intensity cutoff of fragment ion	30 counts
Relative intensity cutoff of fragment ion	0.01
m/z tolerance for precursor ion match	20 ppm or 0.004 Da for m/z < 200 Da
Maximum/minimum RT tolerance for clustering MS2	20 s and 10 s
Maximum/minimum mobility tolerance for clustering MS2	0.030 and 0.015 V·s/cm^2
3D distance cutoff in HCA	1
**# The bottom-up assembly algorithm for 4D peak detection**	
m/z tolerance for MS1 data point search	20 ppm or 0.004 Da for m/z < 200 Da
Mobility range to reconstruct ion mobilogram	0.1 V·s/cm^2
Frame range to reconstruct ion chromatogram	30 frames
Peak span of EIM and EIC	13 points and 11 points
RT difference tolerance of detected EIC apex and precursor RT	10 s
Mobility difference tolerance of detected EIC apex and precursor RT	0.015 V·s/cm^2
Frame range for signal integration	5 frames
Mobility range for signal integration	0.015 V·s/cm^2
Signal-to-noise ratio cutoff	3
Normalized standard noise cutoff	0.35
**# 4D peak alignment and grouping**	
m/z tolerance for landmark match	20 ppm or 0.004 Da for m/z < 200 Da
RT tolerance for landmark match	30 s
CCS tolerance for landmark match	2%
Dot-product cutoff for MS2 spectral match for landmark match	0.8
m/z bin size	0.015 Da
Mobility bin size	0.015 V·s/cm^2
Bandwidth of gaussian smoothing kernel to apply to the peak density chromatogram	5 s
Minimum fraction of samples necessary in at least one of the sample groups	0.5
Frame range for gap filling	5 frames
Mobility range for gap filling	0.015 V·s/cm^2
m/z tolerance for gap filling	20 ppm or 0.004 Da for m/z < 200 Da
**# Multi-dimensional match for metabolite identification**	
m/z tolerance for MS1 match	20 ppm or 0.004 Da for m/z < 200 Da
Maximum/minimum tolerance for RT match	90 s and 30 s
Maximum/minimum tolerance for CCS match	6% and 3%
Dot-product cutoff for MS2 spectral match	0.8
Weight of RT match	0.2
Weight of CCS match	0.4
Weight of MS2 spectral match	0.4
Combined score cutoff	0.6

Supplementary Table 2. Parameter set to run Me4DX (PASEF-DDA, precursor ion list).

Parameter	Value
**# The bottom-up assembly algorithm for 4D peak detection**	
m/z tolerance for MS1 data point search	20 ppm or 0.004 Da for m/z < 200 Da
Mobility range to reconstruct ion mobilogram	0.1 V·s/cm^2
Frame range to reconstruct ion chromatogram	30 frames
Peak span of EIM and EIC	13 points and 11 points
RT difference tolerance of detected EIC apex and precursor	15 s
Mobility difference tolerance of detected EIC apex and precursor	0.015 V·s/cm^2
Frame range for signal integration	5 frames
Mobility range for signal integration	0.015 V·s/cm^2
Signal-to-noise ratio cutoff	3
Normalized standard noise cutoff	0.35
**# MS2 spectral assignment**	
m/z tolerance	20 ppm or 0.004 Da for m/z < 200 Da
RT tolerance	10 s
Mobility tolerance	0.02 V·s/cm^2

Supplementary Table 3. Parameter set to run Me4DX (PASEF-DIA, precursor ion list).

Parameter	Value
**# The bottom-up assembly algorithm for 4D peak detection**	
m/z tolerance for MS1 data point search	20 ppm or 0.004 Da for m/z < 200 Da
Mobility range to reconstruct ion mobilogram	0.1 V·s/cm^2
Frame range to reconstruct ion chromatogram	30 frames
Peak span of EIM and EIC	13 points and 7 points
RT difference tolerance of detected EIC apex and precursor	30 s
Mobility difference tolerance of detected EIC apex and precursor	0.02 V·s/cm^2
Frame range for signal integration	5 frames
Mobility range for signal integration	0.015 V·s/cm^2
Signal-to-noise ratio cutoff	3
Normalized standard noise cutoff	0.35
**# MS2 spectral extraction**	
m/z tolerance to find continuous signals	20 ppm or 0.004 Da for m/z < 200 Da
Mobility tolerance to extract MS2 signals	0.01 V·s/cm^2
Minimal points of continuous signals	2

Supplementary Table 4. Parameter set to run Me4DX (IM-AIF, precursor ion list).

Parameter	Value
**# The bottom-up assembly algorithm for 4D peak detection**	
m/z tolerance for MS1 data point search	20 ppm or 0.004 Da for m/z < 200 Da
DT range to reconstruct ion mobilogram	2 ms
Frame range to reconstruct ion chromatogram	20 frames
Peak span of EIM and EIC	9 points and 9 points
RT difference tolerance of detected EIC apex and precursor	30 s
Mobility difference tolerance of detected EIC apex and precursor	2 ms
Frame range for signal integration	5 frames
DT range for signal integration	0.5 ms
Signal-to-noise ratio cutoff	3
Normalized standard noise cutoff	0.35
**# MS2 spectral extraction**	
m/z tolerance to find continuous signals	20 ppm or 0.004 Da for m/z < 200 Da
DT tolerance to extract MS2 signals	0.5 ms
Minimal points of continuous signals	2

Supplementary Table 5. Parameter set to run MS-DIAL.

Parameter	Value	Parameter name	Value
**# Data collection parameters**		**# Adduct ion setting**	
Retention time range	0-12 min	Adduct	[M+H]+ / [M-H]-
Mass range	20-1300 Da	**# Alignment parameters setting**	
**# Centroid parameters**		Retention time tolerance	0.05 min
MS1 tolerance	0.01 Da	MS1 tolerance	0.015 Da
MS2 tolerance	0.01 Da	Retention time factor	0.5
**#Isotope recognition**		MS1 factor	0.5
Maximum charged number	1	Peak count filter	0%
**# Peak detection parameters**		N% detected in at least one group	50%
Smoothing method	LinearWeightedMovingAverage	Keep identified and annotated metabolites	TRUE
Smoothing level	3 scans	Keep removable features and assign the tag for checking	TRUE
Minimum peak width	5 scans	Gap filling by compulsion	TRUE
Minimum peak height	300 amplitude	**# Ion mobility**	
**# Peak spotting parameters**		Mobility type	Tims
Mass slice width	0.1 Da	Accumulated RT range	0.2 min
**# Deconvolution parameters**		CCS search Tolerance	10 $Å^2$
Sigma window value	0.5	Use CCS for identification scoring	FALSE
MS2Dec amplitude cut off	30 amplitude	Use CCS for identification filtering	TRUE
Exclude after precursor	TRUE	Mobility axis alignment tolerance	0.02 $V{\cdot}s/cm^2$
Keep isotope until	0.5 Da		
Keep original precursor isotopes	FALSE		

Note: The parameters kept the same for PASEF-DDA, PASEF-DIA and IM-AIF data processing except modifying mass ranges according to the data acquisition.

Supplementary Table 6. Parameter set to run MetaboScape.

**Parameter**	**Value**
# **Processing Workflow Result Filter Method**	
Min number of features for extraction	3 (50% in 6 replicates)
Min number of Features for result	3 (50% in 6 replicates)
# **T-ReX 4D Workflow Method**	
Feature signal	Area
Minimum 4D peak size	50 points
Intensity threshold	100 counts
Enable Recursive Feature Extraction	TRUE
Minimum 4D peak size (recursive)	5 points
Retention time range	0-12 min
Mass range	20-1300 Da
MS/MS imported method	Maxsum
Group by collision energy	TRUE
# **Ion Deconvolution Parameter**	
EIC correlation	0.8
Primary ion	[M+H]+ / [M-H]-
Split features if isomers detected	TRUE

Note: The parameters kept the same for PASEF-DDA and PASEF-DIA data processing.

Supplementary Note 1. Instruction to perform metabolite annotation with a user-inputted library in Met4DX.

Met4DX supports metabolite annotation with a user-inputted library in .msp format. Two .msp files should be prepared to performed level 1/2 annotation and level 3 annotation, respectively. The demo files are provided in GitHub (https://github.com/ZhuMetLab/Met4DX). For level 1/2 annotation, in the msp file, each compound must contain information listed below. The LABID must be nonredundant. RAW_ID is the source of the compound in the chemical database. LEVEL indicates the source of MS2 spectrum, of which 1 and 2 indicate that the MS2 spectrum was from experimental acquisition and the external library, respectively. PRECURSORMZ is the extract mass of the compound. FORMULA, SMILES, INCHI and INCHIKEY record the structural information of the compound. INCHIKEY1 is the first segment of INCHIKEY. KINGDOM, SUPERCLASS, CLASS and SUBCLASS are the chemical classes defined by ClassyFire. rt_OTHER records the RT of the compound and ccs_ADDUCT are the CCS values of different adduct forms. It is worth noting that MS2 spectra in positive and negative modes must be recorded in separated msp files and indicated in the ION_MODE. The MS2 spectrum is recorded with *m/z* and intensity of the specific fragment ion.

```
LABID: m4x_000019
RAW_ID: C00021
NAME: S-Adenosyl-L-homocysteine
LEVEL: 1
ION_MODE: negative
FORMULA: C14H20N6O5S
PRECURSORMZ: 384.1216
SMILES: C1=NC2=C(C(=N1)N)N=CN2[C@H]3[C@@H]([C@@H]([C@H](O3)CSCC[C@@H](C(=O)O)N)O)O
INCHI: InChI=1S/C14H20N6O5S/c15-6(14(23)24)1-2-26-3-7-9(21)10(22)13(25-7)20-5-19-8-11(16)1
INCHIKEY: ZJUKTBDSGOFHSH-WFMPWKQPSA-N
INCHIKEY1: ZJUKTBDSGOFHSH
KINGDOM: Organic compounds
SUPERCLASS: Nucleosides, nucleotides, and analogues
CLASS: 5'-deoxyribonucleosides
SUBCLASS: 5'-deoxy-5'-thionucleosides
rt_OTHER: 376.14000
ccs_[M+H]+: 185.0018
ccs_[M+Na]+: 187.8074
ccs_[M+NH4]+: 187.1839
ccs_[M-H2O+H]+: 182.6271
ccs_[M-H]-: 181.9391
ccs_[M+Na-2H]-: 181.8802
ccs_[M+HCOO]-: 181.9527
Num Peaks: 8
74.99082 0.0270922987754137
86.99071 0.0256875423522131
107.035525 0.0152467836676877
116.01765 0.0144354312166821
134.04717 1
135.04987 0.0155968542682659
176.039835 0.0144472805662683
188.03897 0.0136894141047767
```

Example for the msp file used for level1/2 annotation

The msp file for level 3 annotation contains similar information. The LEVEL is 3 while the MS2 spectrum is recorded as 0 in this file.

```
LABID: m4x_000049
RAW_ID: C00061
NAME: FMN
LEVEL: 3
ION_MODE: negative
FORMULA: C17H21N4O9P
PRECURSORMZ: 456.1046
SMILES: CC1=CC2=C(C=C1C)N(C3=NC(=O)NC(=O)C3=N2)C[C@@H]([C@@H]([C@@H](COP(=O)(O)O)O)O)O
INCHI: InChI=1S/C17H21N4O9P/c1-7-3-9-10(4-8(7)2)21(15-13(18-9)16(25)20-17(26)19-15)5-11(2
INCHIKEY: FVTCRASFADXXNN-SCRDCRAPSA-N
INCHIKEY1: FVTCRASFADXXNN
KINGDOM: Organic compounds
SUPERCLASS: Nucleosides, nucleotides, and analogues
CLASS: Flavin nucleotides
SUBCLASS: NA
rt_HILIC: 404.49000
ccs_[M+H]+: 198.8495
ccs_[M+Na]+: 201.2582
ccs_[M+NH4]+: 200.7241
ccs_[M-H2O+H]+: 196.8001
ccs_[M-H]-: 194.0855
ccs_[M+Na-2H]-: 194.4941
ccs_[M+HCOO]-: 195.0826
Num Peaks: 1
0 0
```

Example for the msp file used for level 3 annotation

Users could use the function “IdentifyPeaks_input_library” in Met4DX to use the inputted library for metabolite annotations. “lib_file” is the path to level1/2 msp file, while “level3_lib_file” is the path to level3 msp file.

```
IdentifyPeaks_input_library(tims_data,
                            param,
                            match_para,
                            combine_para,
                            rt_exp_file = NULL,
                            rt_ref_file = NULL,
                            lib_file = 'level1_2.msp', # path to level1_2 msp file
                            level3_lib_file = 'level3_.msp', # path to level3 msp file
                            demo_mode = FALSE)
```
