# Supplementary material for: A mass spectrum-oriented computational method for ion mobility-resolved untargeted metabolomics
Source: Nat Commun. 2023 Mar 31;14:1813. doi: 10.1038/s41467-023-37539-0 (PMC10066191; doi:10.1038/s41467-023-37539-0)
Supplement: Supplementary file 3 — Description of Additional Supplementary Files [file 41467_2023_37539_MOESM3_ESM.docx]

File Name: Supplementary Data 1

Description: 4D features detected by Met4DX in biological samples.

File Name: Supplementary Data 2

Description: 4D features detected by MS-DIAL in biological samples.

File Name: Supplementary Data 3

Description: 4D features detected by MetaboScape in biological samples.

File Name: Supplementary Data 4

Description: Peak fidelity of 4D features in Met4DX.

File Name: Supplementary Data 5

Description: Peak fidelity of 4D features in MS-DIAL.

File Name: Supplementary Data 6

Description: Peak fidelity of 4D features in MetaboScape.

File Name: Supplementary Data 7

Description: Peak area over the dilution series of 20 natural products in NIST human urine samples.

File Name: Supplementary Data 8

Description: Metabolite annotation in biological samples.

File Name: Supplementary Data 9

Description: Precursor ion list in Met4DX.

File Name: Supplementary Data 10

Description: 4D features detected by Met4DX in NIST human urine with the precursor list (PASEF-DDA).

File Name: Supplementary Data 11

Description: 4D features detected by different software tools in NIST human urine (PASEF-DIA).

File Name: Supplementary Data 12

Description: 4D features detected by different software tools in NIST human urine (IM-AIF).
