# Supplementary material for: A mass spectrum-oriented computational method for ion mobility-resolved untargeted metabolomics
Source: Nat Commun. 2023 Mar 31;14:1813. doi: 10.1038/s41467-023-37539-0 (PMC10066191; doi:10.1038/s41467-023-37539-0)
Supplement: Supplementary file 16 — Reporting Summary [file 41467_2023_37539_MOESM16_ESM.pdf]

nature portfolio

Corresponding author(s): Zheng-Jiang Zhu

Last updated by author(s): Mar 7, 2023

Reporting Summary

Nature Portfolio wishes to improve the reproducibility of the work that we publish. This form provides structure for consistency and transparency in reporting. For further information on Nature Portfolio policies, see our Editorial Policies and the Editorial Policy Checklist.

Statistics

For all statistical analyses, confirm that the following items are present in the figure legend, table legend, main text, or Methods section.

n/a	Confirmed	
☐	☒	The exact sample size (n) for each experimental group/condition, given as a discrete number and unit of measurement
☐	☒	A statement on whether measurements were taken from distinct samples or whether the same sample was measured repeatedly
☒	☐	The statistical test(s) used AND whether they are one- or two-sided<br>*Only common tests should be described solely by name; describe more complex techniques in the Methods section.*
☒	☐	A description of all covariates tested
☒	☐	A description of any assumptions or corrections, such as tests of normality and adjustment for multiple comparisons
☐	☒	A full description of the statistical parameters including central tendency (e.g. means) or other basic estimates (e.g. regression coefficient) AND variation (e.g. standard deviation) or associated estimates of uncertainty (e.g. confidence intervals)
☒	☐	For null hypothesis testing, the test statistic (e.g. F, t, r) with confidence intervals, effect sizes, degrees of freedom and P value noted<br>*Give P values as exact values whenever suitable.*
☒	☐	For Bayesian analysis, information on the choice of priors and Markov chain Monte Carlo settings
☒	☐	For hierarchical and complex designs, identification of the appropriate level for tests and full reporting of outcomes
☐	☒	Estimates of effect sizes (e.g. Cohen's d, Pearson's r), indicating how they were calculated

Our web collection on statistics for biologists contains articles on many of the points above.

Software and code

Policy information about availability of computer code

Data collection	LC-IM-MS data acquisition were performed with timsControl (version 2.0, Bruker Daltonics, Bremen, Germany) and MassHunter Workstation Data Acquisition Software (version B.08.00, Agilent Technologies, USA).
Data analysis	The source code of Met4DX was provided in GitHub [https://github.com/ZhuMetLab/Met4DX] and Zenodo [https://doi.org/10.5281/zenodo.7701165]. (version 2.0.0). Other software tools: MS-DIAL (v 4.60 for DDA data analysis and 4.90 for DIA data analysis); MetaboScape (v2022b, Bruker Daltonics, Bremen, Germany); DataAnalysis (v5.2, Bruker Daltonics, Bremen, Germany); ProteoWizard (version 3.0.20360); R (v 4.2.1); opentimsr (v 1.0.13); MS-FINDER (v 3.24).

For manuscripts utilizing custom algorithms or software that are central to the research but not yet described in published literature, software must be made available to editors and reviewers. We strongly encourage code deposition in a community repository (e.g. GitHub). See the Nature Portfolio guidelines for submitting code & software for further information.

Data

Policy information about availability of data

All manuscripts must include a data availability statement. This statement should provide the following information, where applicable:

- Accession codes, unique identifiers, or web links for publicly available datasets
- A description of any restrictions on data availability
- For clinical datasets or third party data, please ensure that the statement adheres to our policy

All raw data files of biological samples acquired by TIMS with PASEF-DDA can be assessed by National Omics Data Encyclopedia [Project ID: OEP003701; https://

www.biosino.org/node/project/detail/OEP003701] and Zenodo [https://doi.org/10.5281/zenodo.7215544]. Raw data files of NIST human urine sample acquired by TIMS with PASEF-DIA and DTIM-MS with IM-AIF can be assessed by National Omics Data Encyclopedia [Project ID: OEP003846; https://www.biosino.org/node/project/detail/OEP003846]. KEGG database was accessed on 7th March, 2017 [https://www.genome.jp/kegg/]. HMDB database was accessed on 8th November, 2021 [https://hmdb.ca/]. Source data are provided with this paper.

Human research participants

Policy information about studies involving human research participants and Sex and Gender in Research.

Reporting on sex and gender	No human participant is involved in this study.
Population characteristics	No human participant is involved in this study.
Recruitment	No human participant is involved in this study.
Ethics oversight	No human participant is involved in this study.

Note that full information on the approval of the study protocol must also be provided in the manuscript.

Field-specific reporting

Please select the one below that is the best fit for your research. If you are not sure, read the appropriate sections before making your selection.

☒ Life sciences ☐ Behavioural & social sciences ☐ Ecological, evolutionary & environmental sciences

For a reference copy of the document with all sections, see nature.com/documents/nr-reporting-summary-flat.pdf

Life sciences study design

All studies must disclose on these points even when the disclosure is negative.

Sample size	Datasets acquired in this study were performed on six technical replicates to demonstrate software applicability instead of generating biological results. Six replicates were chosen because they are the commonly used number for software development towards data processing.
Data exclusions	No samples were excluded from analysis in this study.
Replication	Each sample in the manuscript were measured with 6 technical replicates. All attempts at replication were successful.
Randomization	The randomization is not required, because this study only included technical replicates.
Blinding	It did not require the use of blinding, because these datasets were used to demonstrate software applicability instead of generating biological results.

Reporting for specific materials, systems and methods

We require information from authors about some types of materials, experimental systems and methods used in many studies. Here, indicate whether each material, system or method listed is relevant to your study. If you are not sure if a list item applies to your research, read the appropriate section before selecting a response.

Materials & experimental systems

n/a	Involved in the study	
☒	☐	Antibodies
☐	☒	Eukaryotic cell lines
☒	☐	Palaeontology and archaeology
☐	☒	Animals and other organisms
☒	☐	Clinical data
☒	☐	Dual use research of concern

Methods

n/a	Involved in the study	
☒	☐	ChIP-seq
☒	☐	Flow cytometry
☒	☐	MRI-based neuroimaging

Eukaryotic cell lines

Policy information about cell lines and Sex and Gender in Research

Cell line source(s)	The 293T cell line was obtained from ATCC with Product No. CRL-2925.
Authentication	Authentication was done using STR (Short Tandem Repeat) analysis.
Mycoplasma contamination	The cell lines were not tested for mycoplasma contamination.
Commonly misidentified lines (See ICLAC register)	No commonly misidentified cells were used.

Animals and other research organisms

Policy information about studies involving animals; ARRIVE guidelines recommended for reporting animal research, and Sex and Gender in Research

Laboratory animals	Mouse (c57BL/6J, male, 6-week old);
Wild animals	The study did not involve animals collected from the filed.
Reporting on sex	The mouse liver and brain datasets were used to demonstrate software applicability instead of generating biological results. So the sex is not reported.
Field-collected samples	The study did not involve animals collected from the filed.
Ethics oversight	They study did not need ethic oversight.

Note that full information on the approval of the study protocol must also be provided in the manuscript.
